# Hidden Hunger in the Age of Abundance: The Nutritional Pitfalls of Modern Staple Crops

**DOI:** 10.1002/fsn3.4610

**Published:** 2025-01-02

**Authors:** Hilal Yilmaz, Abdurrahim Yilmaz

**Affiliations:** ^1^ Izmit Vocational High School, Plant and Animal Production Program Kocaeli University Kocaeli Turkey; ^2^ Department of Field Crops, Faculty of Agriculture Bolu Abant Izzet Baysal University Bolu Turkey

**Keywords:** cereals, green revolution, legumes, modern agriculture, nutrient deficiencies

## Abstract

Hidden hunger, characterized by micronutrient deficiencies despite adequate caloric intake, affects over 2 billion people globally, primarily due to deficits in iron, vitamin A, and iodine. This phenomenon underscores a critical paradox in global food security: the Green Revolution, which significantly increased crop production through high‐yielding varieties (HYVs) of staple crops, has simultaneously contributed to widespread nutritional deficiencies. This article examines the dual legacy of the Green Revolution, exploring how its emphasis on yield over nutritional quality has led to decreased concentrations of essential micronutrients in staple crops, exacerbating hidden hunger. The extensive use of synthetic fertilizers, while boosting crop yields, has resulted in environmental degradation and economic burdens for smallholder farmers. Additionally, the shift towards dietary monoculture has reduced agricultural biodiversity and increased the prevalence of diet‐related non‐communicable diseases. Through diverse case studies from India, Zambia, Guatemala, the Philippines, Brazil, Mexico, and Ethiopia, this article illustrates various strategies to combat hidden hunger, including biofortification, multisectoral approaches, and sustainable agricultural practices. This article highlights the necessity for a multifaceted approach that integrates improved agricultural practices, dietary diversity, and supportive policies to enhance food security and public health. By addressing both caloric and nutritional needs, this comprehensive strategy aims to build resilient food systems that ensure a sustainable agricultural future.

## Graphical abstract

The Green Revolution significantly boosted global food production but, over time, led to nutrient imbalances in staple foods, amplifying the risk of hidden hunger. This article explores the decline in micronutrient concentrations in today's staple crops and their impact on malnutrition. To tackle these challenges, sustainable agricultural practices and biofortification strategies are crucial for ensuring both food security and nutritional sufficiency.

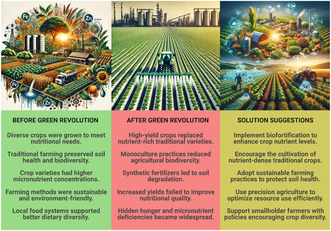

## 1 Introduction

Modern agriculture is undergoing rigorous transformations due to the environmental challenges posed by the rapid growth of the world's population, compelling efforts to ensure global food security and maintain sustainability (Yilmaz et al. 2023). There is an urgent necessity to transform agricultural management and production practices to secure economic viability and bolster farmers' livelihoods (Yılmaz 2023). Boosting yield per unit area is crucial for agriculturally dependent countries to satisfy their food and nutritional requirements and sustain their economic stability (Yılmaz et al. 2022). However, increased production alone does not ensure improved nutritional outcomes; the quality of the food produced is equally important (Pingali 2015). In this context, one of the most pressing issues in modern agriculture is not just the quantity of food produced, but its quality (Khoury et al. 2014).

Hidden hunger, a term increasingly used in public health discourse, refers to micronutrient deficiencies that occur even when caloric intake is sufficient. Despite an apparent abundance of food, millions of individuals globally suffer from deficiencies in essential vitamins and minerals, leading to severe health problems (Ritchie and Roser 2021; Bailey, West Jr, and Black 2015). According to a report by the World Health Organization (WHO 2021), over 2 billion individuals worldwide suffer from hidden hunger, predominantly due to deficiencies in iron, vitamin A, and iodine. This paradox of hidden hunger highlights a critical issue: while food quantity has improved globally, quality has not kept pace, resulting in widespread micronutrient deficiencies (Fanzo et al. 2018; Development Initiatives 2021).

To understand the root of this problem, it is essential to revisit the historical developments in agriculture. The Green Revolution, which began in the mid‐20th century, played a pivotal role in transforming agricultural practices and boosting food production (Pingali 2012). The introduction of high‐yielding varieties (HYVs) of staple crops such as wheat and rice, coupled with increased use of synthetic fertilizers and irrigation, significantly increased food production and helped combat hunger in many developing regions (Evenson and Gollin 2003; Gollin, Hansen, and Wingender 2021). However, research has shown that modern varieties of staple crops tend to have lower concentrations of essential micronutrients compared to their traditional counterparts (Bouis and Saltzman 2017; Bouis et al. 2019). This focus on high‐yield crops often led to the neglect of traditional, nutrient‐rich crops, contributing to a reduction in dietary diversity (Pingali 2012; Müller and Krawinkel 2005).

Thus, the paradox of hidden hunger in the context of abundant food production underscores the complex relationship between agricultural practices and nutritional outcomes. While the Green Revolution succeeded in addressing the immediate threat of hunger, it inadvertently contributed to a different form of malnutrition (Shetty 2018; Graham et al. 2007). This shift necessitates a re‐evaluation of agricultural priorities, emphasizing not just the quantity but also the nutritional quality of the food produced (Khoury et al. 2014). As the world continues to grapple with malnutrition in its various forms, addressing hidden hunger becomes an essential component of global food security and public health strategies (Burchi, Fanzo, and Frison 2011; Smith and Haddad 2015). To further illustrate these key issues, the figures presented throughout this review have been meticulously designed to offer visual representations of the complex relationship between modern agricultural transformations and nutritional outcomes, particularly hidden hunger. These visuals aim to reinforce the discussion, providing a comprehensive understanding of how agricultural practices have influenced both food security and nutritional quality.

## 2 The Green Revolution: A Double‐Edged Sword

The Green Revolution, a major agricultural transformation that began in the 1940s and continued through the late 1960s, aimed to increase food production to combat global hunger (Pingali 2012; Evenson and Gollin 2003). This movement led to the development and widespread adoption of HYVs of staple crops like wheat and rice, alongside advances in irrigation, synthetic fertilizers, and pesticides (Gollin, Hansen, and Wingender 2021). These innovations significantly boosted crop yields, with wheat and rice production in developing countries more than doubling by the end of the 20th century (Foley et al. 2011). The increased yields were instrumental in preventing famines and significantly reducing hunger in many parts of the world (Gollin, Hansen, and Wingender 2021).

However, the Green Revolution's focus on maximizing yield often came at the expense of nutritional quality and environmental sustainability (Pingali 2012). One significant unintended consequence has been the decline in the nutritional content of staple crops. Research indicates that HYVs of wheat and rice have lower concentrations of essential micronutrients compared to traditional strains (Calayugan et al. 2020; Majumder, Datta, and Datta 2019; Saquee et al. 2023). This reduction in micronutrient density has exacerbated the issue of hidden hunger, where populations meet their caloric needs but suffer from deficiencies in critical vitamins and minerals (Fanzo et al. 2018).

The Green Revolution has had significant environmental consequences, primarily due to the widespread use of synthetic fertilizers and pesticides. These practices have resulted in soil degradation, water pollution, and reduced biodiversity (Galloway et al. 2003; Tilman et al. 2011). Nitrogen‐based fertilizers, while increasing crop yields, have been linked to groundwater contamination and harmful algal blooms, posing significant ecological and health risks (Mueller et al. 2012). Additionally, the reliance on monoculture farming practices has made crops more vulnerable to pests and diseases, necessitating even greater use of chemical inputs (Khoury et al. 2014).

**FIGURE 1:**
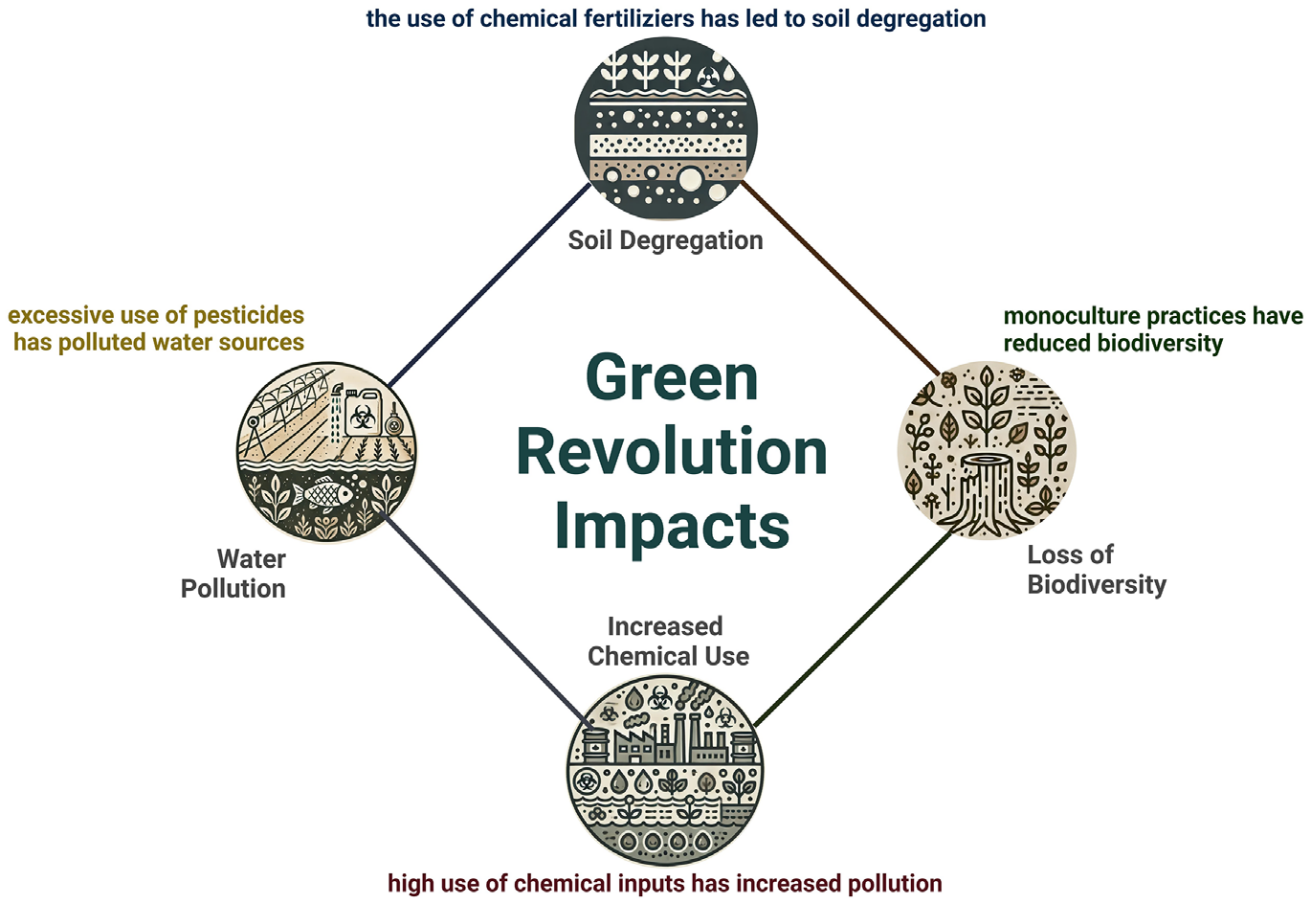
Environmental impacts of the green revolution.

The shift towards monoculture and high‐yielding crops has also reduced agricultural biodiversity, with many traditional, nutritionally rich crops being abandoned (Lawson et al. 2018). This loss of crop diversity has not only impacted nutrition but also reduced the resilience of agricultural systems to environmental stresses such as climate change (Lin 2011). The focus on a few major crops has led to dietary monoculture, where diets become less varied and more reliant on starchy staples, contributing to an increase in diet‐related diseases (Johns and Eyzaguirre 2006). Figure 1 illustrates the environmental degradation resulting from the Green Revolution, emphasizing the interconnectedness of soil degradation, water pollution, loss of biodiversity, and increased chemical use. These impacts directly result from the intensified focus on a few high‐yield crops, leading to reduced agricultural diversity and resilience to environmental stressors.

Despite the Green Revolution's success in increasing food production and reducing immediate hunger, it has underscored the necessity for a more holistic approach to agriculture. Addressing the challenges of nutritional quality, environmental sustainability, and agricultural biodiversity is crucial for future food security (Pingali 2012; Burchi, Fanzo, and Frison 2011). Integrating sustainable farming practices, promoting dietary diversity, and focusing on the nutritional value of crops are essential steps towards a more balanced and resilient agricultural system (Ekesa, Walingo, and Abukutsa‐Onyango 2008; Garnett et al. 2013; Campos et al. 2019; Dhillon and Moncur 2023).

## 3 Decreased Nutritional Quality of Staple Crops

The nutritional quality of staple crops such as wheat and rice has seen a significant decline over the past few decades (Paine et al. 2005; Smith, Golden, and Myers 2017; Table 1). HYVs, bred primarily for their productivity, often have lower concentrations of essential nutrients compared to traditional varieties (Muthayya et al. 2013). Modern wheat varieties, for instance, have 19%–28% lower concentrations of minerals such as zinc, iron, and magnesium compared to older varieties (Fanzo et al. 2018). Similarly, Fan et al. (2008) reported a significant decrease in the concentration of important minerals in wheat grains over the last 160 years. At the same time, they highlighted that the iron content in modern wheat varieties has significantly decreased compared to traditional varieties. Numerous studies have reported that, in recent years, HYVs of wheat, maize, and rice tend to exhibit reductions in essential micronutrients, such as iron, zinc, and protein, compared to traditional varieties (Liu et al. 2007; Pahlavan‐Rad and Pessarakli 2009; Anandan et al. 2011; Bouis and Saltzman 2017; Garcia‐Oliveira et al. 2018; Garg et al. 2018; Rehman et al. 2018; Majumder, Datta, and Datta 2019; Calayugan et al. 2020; Avnee et al. 2023; Gedil et al. 2024; Raut et al. 2024; Sen, Kumar, and Janeja 2024).

Iron deficiency can cause anemia, leading to fatigue, weakened immune function, and impaired cognitive development. The WHO reports that anemia affects approximately 1.62 billion people globally, particularly impacting children and pregnant women (World Health Organization 2020).

**FIGURE 2:**
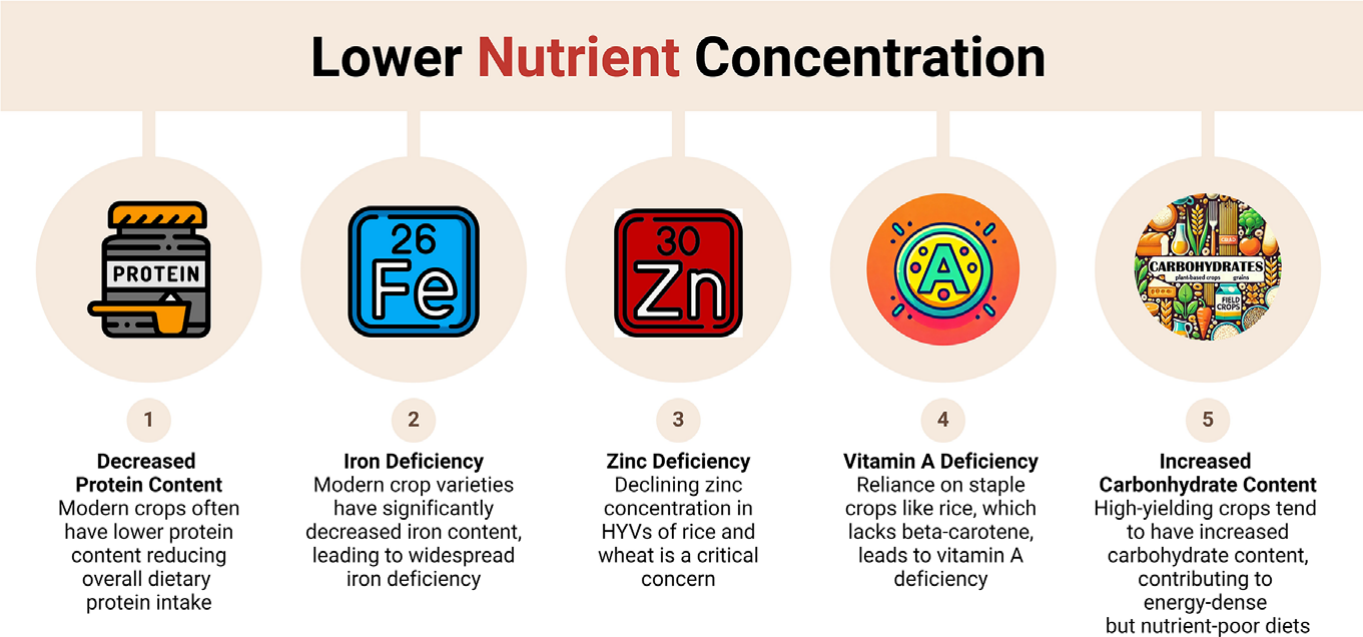
Low nutrient concentrations in a transformed agricultural landscape (HVYs: high‐yielding varieties).

Zinc deficiency remains a critical concern linked to staple crops. Zinc concentration in HYVs of rice and wheat has declined over the past few decades. Zinc is essential for immune function, wound healing, and DNA synthesis. Zinc deficiency can result in growth retardation, delayed sexual maturation, and increased susceptibility to infections. Khan and Malik (2022) reported that zinc deficiency affects nearly 17% of the global population, predominantly in regions where rice and wheat are dietary staples. Vitamin A deficiency, primarily due to the reliance on staple crops like rice, poses severe public health challenges. White rice, the most consumed form, lacks beta‐carotene, a precursor to vitamin A. This deficiency can lead to night blindness and increase the risk of severe infections and mortality among children. According to Stevens et al. (2015), an estimated 190 million preschool‐aged children globally are affected by vitamin A deficiency. Figure 2 emphasizes the significant decline in essential nutrients like protein, iron, zinc, and vitamin A in high‐yielding crop varieties, while also showing the increased carbohydrate content. This imbalance contributes to energy‐dense but nutrient‐poor diets, exacerbating the nutritional challenges associated with modern agriculture. The visual highlights the complexity of these changes in crop composition and their impacts on global health.

Several mechanisms contribute to the reduction in nutritional quality in HYVs. One primary factor is the “dilution effect,” where increased carbohydrate content in high‐yield crops leads to a proportional decrease in protein and micronutrient concentrations (Fan et al. 2008). This effect is particularly evident in cereals like wheat and rice, which form the dietary foundation for millions of people worldwide (Fahad et al. 2017; Nsafon, Lee, and Huh 2020;). As yield per hectare increases, the nutrient density of the grains often decreases (Welch and Graham 1999). Furthermore, selective breeding for traits such as pest resistance and faster growth cycles has inadvertently deprioritized nutritional quality (Muthayya et al. 2013).

Environmental factors also play a significant role in the declining nutritional quality of staple crops. Increased levels of atmospheric carbon dioxide (CO_2_), a result of climate change, have been shown to reduce the concentration of essential nutrients in crops (Dietterich et al. 2015; Ebi et al. 2021). Elevated CO_2_ levels can decrease the protein, iron, and zinc content in wheat and rice by 5%–10% (Zhu et al. 2018). Additionally, modern agricultural practices, such as the extensive use of synthetic fertilizers, have led to soil degradation and reduced soil fertility, further impacting the nutrient content of crops (Galloway et al. 2003; Mueller et al. 2012).

The bioavailability of nutrients is another crucial factor influencing the nutritional value of crops. Even when crops contain adequate amounts of micronutrients, the presence of antinutritional factors like phytates can inhibit nutrient absorption in the human body (Welch and Graham 1999). Modern breeding practices often overlook these aspects, focusing instead on yield and disease resistance (Bouis, Saltzman, and Birol 2019). Efforts to biofortify crops—enhancing their nutrient content through conventional breeding or genetic modification—are ongoing but face challenges in ensuring that these nutrients are bioavailable and effectively utilized by the human body (Bouis and Saltzman 2017). While biofortification offers a promising strategy for addressing micronutrient deficiencies, its success heavily depends on the participation of agricultural stakeholders. Incentives such as government subsidies, financial support for farmers, and market access can play a vital role in encouraging the adoption of biofortified crops. For instance, policy interventions that offer financial benefits for growing nutrient‐enriched crops can motivate farmers to shift production practices. Additionally, consumer demand for healthier, nutrient‐dense foods could further drive agricultural participation (Pingali 2015). Incorporating such incentives into national agricultural programs could significantly enhance the impact of biofortification initiatives.

The decline in nutritional quality of staple crops has significant public health implications. Hidden hunger, characterized by micronutrient deficiencies despite adequate caloric intake, is a growing concern globally (Ritchie and Roser 2021). This issue is exacerbated in developing countries where diets heavily rely on a few staple crops, making populations particularly vulnerable to nutrient deficiencies. Addressing this problem requires a multifaceted approach, integrating agronomic practices that enhance soil health, breeding strategies that prioritize nutritional quality, and policies that promote dietary diversity (Khoury et al. 2014; Fanzo et al. 2018). Biofortification and food fortification are two key strategies being employed to address these deficiencies. Biofortification involves breeding crops to increase their nutrient content, thereby enhancing the nutritional value of staple foods. A study by Saltzman et al. (2013) found that biofortified crops can significantly improve nutrient intake and health outcomes in populations that rely heavily on staple foods. Food fortification, the addition of essential nutrients to processed foods, is another effective strategy. Fortifying flour with iron and folic acid, for example, has been shown to reduce the prevalence of anemia and neural tube defects (Darnton‐Hill et al. 2005; Kancherla et al. 2020). Similarly, fortifying rice with iron, zinc, and vitamin A can help address multiple nutrient deficiencies simultaneously (Muthayya et al. 2013).

While the bioavailability and effective utilization of nutrients are key concerns in biofortification efforts, public acceptance of genetically modified crops has posed significant hurdles. For example, Golden Rice, a genetically modified rice variety developed to address vitamin A deficiency, faced considerable resistance despite its proven efficacy (Tang et al. 2012). Public concerns around genetically modified organisms (GMOs) focus on health, environmental, and ethical considerations. Recent studies highlight public skepticism regarding the long‐term health impacts of GMOs, particularly due to the potential for unforeseen allergenicity or gene transfer (Shen et al. 2022). Environmental worries revolve around the unintended consequences of GM crops, such as their potential to affect biodiversity or lead to increased herbicide use (Antoniou et al. 2023). Ethical debates often focus on corporate control over the food supply and the right to label GM foods (Bacha and Iqbal 2023). Addressing these concerns through transparent communication and regulatory frameworks could help foster greater acceptance and utilization of such technologies, enabling their potential public health benefits to be realized.

The reliance on a limited number of staple crops has created a dietary monoculture that exacerbates nutrient deficiencies. Diversifying agricultural production and encouraging the consumption of a wider variety of foods are critical for improving nutritional outcomes. Integrating legumes, fruits, vegetables, and biofortified crops into diets can help provide a more balanced intake of essential nutrients (Herforth et al. 2020).

## 4 Loss of Agricultural Biodiversity

Before the Green Revolution, agricultural systems worldwide were characterized by high levels of biodiversity, with farmers cultivating a wide array of crops adapted to local conditions and cultural preferences (Khoury et al. 2014). This diversity was essential not only for food security but also for maintaining ecological resilience and providing a range of ecosystem services (Altieri 2018). Traditional farming systems typically included multiple varieties of grains, legumes, vegetables, and fruits, each contributing to a balanced diet and sustainable agricultural practices (Jarvis et al. 2008).

The advent of the Green Revolution in the mid‐20th century brought about significant changes in agricultural practices, emphasizing HYVs of staple crops such as wheat and rice (Pingali 2012). While these HYVs substantially increased food production and helped reduce hunger in many regions, they also led to a significant reduction in crop genetic diversity (Bélanger and Pilling 2019). In India, for example, the introduction of HYVs resulted in the abandonment of numerous traditional rice varieties that had been cultivated for centuries (Eliazer Nelson, Ravichandran, and Antony 2019). This shift has led to the loss of valuable genetic traits crucial for adapting to changing environmental conditions and pest pressures (Van de Wouw et al. 2010).

**FIGURE 3:**
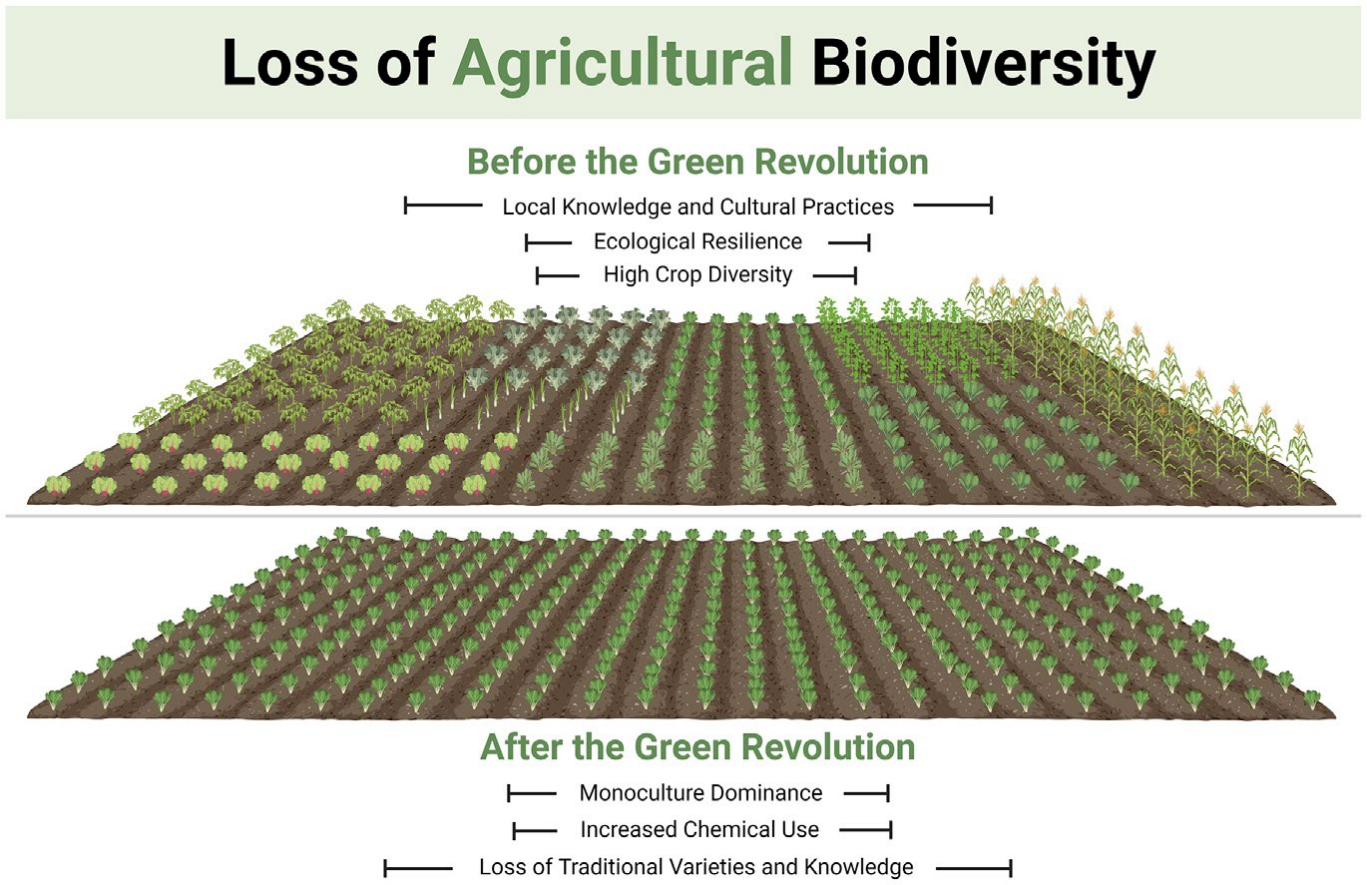
Loss of agricultural biodiversity in the Green Revolution era.

The decline in agricultural biodiversity has profound ecological impacts. Monoculture farming systems, which dominate modern agriculture, are more susceptible to pests and diseases, necessitating increased use of chemical pesticides and fertilizers (Altieri 2018). This not only degrades soil health but also reduces the resilience of agricultural systems to environmental stressors such as climate change (Díaz et al. 2019). Diverse cropping systems significantly reduce pest outbreaks and increase ecosystem stability, underscoring the ecological advantages of preserving agricultural biodiversity (Power 2010). Figure 3 illustrates the transition from diverse agricultural systems to monoculture practices, highlighting the loss of traditional varieties and local knowledge. This visual complements the discussion on how this shift has led to decreased resilience against environmental stressors, increased chemical use, and reduced ecosystem stability.

Several initiatives aim to preserve and restore agricultural biodiversity. One such effort is the establishment of seed banks, which conserve the genetic material of traditional crop varieties. The Svalbard Global Seed Vault in Norway, for example, stores seeds from around the world, providing a safeguard against the loss of genetic diversity (Westengen, Jeppson, and Guarino 2013). Another initiative is the promotion of agroecological farming practices, which integrate biodiversity into agricultural systems to enhance sustainability and resilience (Altieri and Nicholls 2017). These practices include crop rotation, intercropping, and the use of cover crops, all of which can help maintain soil fertility, reduce pest and disease incidence, and improve water management (Kremen and Miles 2012).

Community‐based approaches are also vital for preserving agricultural biodiversity. Farmer‐led conservation initiatives, such as those in the Andean regions of South America, support the cultivation and exchange of traditional crop varieties (Brush 2019). These programs not only help conserve genetic diversity but also strengthen local food systems and cultural heritage. In Africa, the Alliance for a Green Revolution in Africa promotes the use of indigenous crops that are well adapted to local conditions, aiming to improve food security and resilience to climate change.

By integrating traditional knowledge with modern conservation techniques, the resilience and sustainability of agricultural systems can be enhanced in response to global challenges (Altieri 2018). However, while the Green Revolution significantly increased agricultural productivity, it has also led to substantial losses in agricultural biodiversity over the last few decades. This loss poses risks to food security and ecosystem health, underscoring the need for strategies that promote the conservation and sustainable use of crop genetic resources (Khoury et al. 2014).

## 5 The Consequences of Dietary Monoculture

The Green Revolution, while significantly increasing global food production, has also contributed to the phenomenon of dietary monoculture, where diets heavily rely on a few staple crops such as wheat, rice, and maize (Pingali 2012). This shift towards monoculture has led to a reduction in dietary diversity and has significant health implications, particularly concerning nutrient deficiencies (Fanzo et al. 2018). Reliance on a limited number of staple crops can result in insufficient intake of essential vitamins and minerals, leading to conditions such as anemia, stunting, and various micronutrient deficiencies (Graham et al. 2007).

Dietary monoculture is particularly problematic in regions where staple crops dominate daily consumption patterns. In many developing countries, rice and wheat constitute the primary sources of calories, often providing over 60% of the daily energy intake (Pingali 2012). This heavy reliance on a narrow range of food sources can lead to deficiencies in micronutrients such as iron, zinc, and vitamin A, which are not adequately provided by these staples alone (Graham et al. 2007). For instance, the widespread consumption of polished rice, which is low in iron and zinc, has been linked to high rates of anemia and stunting among children in South and Southeast Asia (Muthayya et al. 2013).

Quantitative measures of dietary diversity, such as the Dietary Diversity Score (DDS), provide valuable insights into the correlation between diet variety and health outcomes. A higher DDS is generally associated with better nutritional status and lower prevalence of nutrient deficiencies (Ruel 2003). Studies have shown that populations with low DDS often suffer from multiple micronutrient deficiencies. Children in households with low dietary diversity had significantly higher rates of stunting and wasting compared to those in households with more diverse diets (Arimond and Ruel 2004).

**FIGURE 4:**
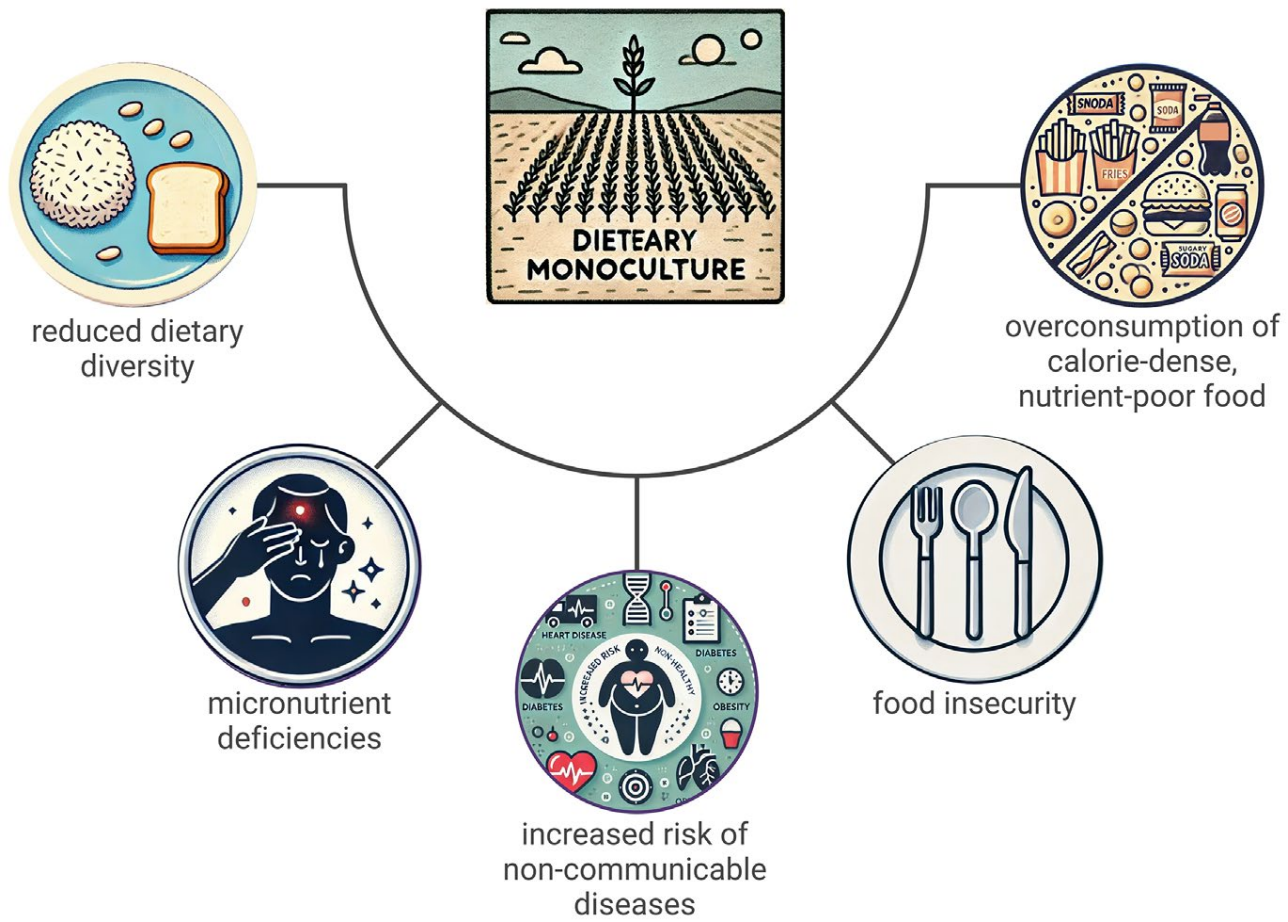
Negative health and nutritional impacts of dietary monoculture.

The health implications of dietary monoculture extend beyond micronutrient deficiencies. Diets lacking diversity are also associated with higher risks of non‐communicable diseases (NCDs) such as diabetes, cardiovascular diseases, and obesity (Micha et al. 2017). The overconsumption of calorie‐dense but nutrient‐poor foods, a common feature of dietary monoculture, contributes to the global rise in NCDs. This trend underscores the importance of promoting dietary diversity to improve both nutritional and overall health outcomes (Tilman and Clark 2014). Figure 4 visually represents the adverse effects of dietary monoculture, emphasizing reduced dietary diversity, increased micronutrient deficiencies, and the overconsumption of calorie‐dense, nutrient‐poor foods. These factors contribute to rising food insecurity and a growing prevalence of NCDs globally.

Addressing the consequences of dietary monoculture requires comprehensive policy and educational efforts to promote dietary diversity. Governments and international organizations play a crucial role in shaping food policies that encourage the production and consumption of a wider variety of nutrient‐dense foods. The Food and Agriculture Organization advocates for the integration of diverse crops into agricultural systems to enhance food security and nutrition (Food and Agriculture Organization of the United Nations 2019). Policies that support the cultivation of traditional and indigenous crops can help diversify diets and improve nutrient intake (Johns and Eyzaguirre 2006).

Educational initiatives are equally important in promoting dietary diversity. Public health campaigns aimed at raising awareness about the benefits of a varied diet can influence consumer behavior and dietary choices (Burlingame and Dernini 2011). Nutrition education programs in schools can teach children about the importance of including a range of foods in their diets, helping to establish healthy eating habits early in life (Food and Agriculture Organization of the United Nations 2019). Community‐based programs that involve local farmers and food producers can also play a pivotal role in promoting dietary diversity by encouraging the cultivation and consumption of diverse crops (Herforth and Ahmed 2015).

In addition to policy and educational efforts, there is a growing movement towards sustainable agricultural practices that support dietary diversity. Agroecology, which emphasizes biodiversity and ecological principles in farming, can enhance the availability of diverse food sources (Altieri 2018). Practices such as crop rotation, intercropping, and agroforestry not only improve soil health and productivity but also provide a variety of foods that contribute to a balanced diet (Kremen and Miles 2012).

Research and development in biofortification also offer promising solutions to the challenges posed by dietary monoculture. For example, recent studies have developed biofortified maize varieties enriched with provitamin A, such as the orange maize varieties studied by Palmer et al. (2016), which have been shown to improve vitamin A intake and status in communities where maize is a staple food. These biofortified crops can play a critical role in improving dietary quality and preventing nutrient deficiencies.

The shift towards dietary monoculture has significant health implications, particularly regarding nutrient deficiencies and the rise of NCDs. Promoting dietary diversity through policy measures, educational initiatives, sustainable agricultural practices, and biofortification is essential to address these challenges. By encouraging the production and consumption of a wide variety of nutrient‐dense foods, it is possible to improve nutritional outcomes and enhance overall health (Food and Agriculture Organization of the United Nations 2019).

## 6 Environmental and Economic Costs of Increased Fertilizer Use

The extensive use of synthetic fertilizers has been integral to the Green Revolution, significantly boosting agricultural yields. However, this reliance has led to substantial economic and environmental costs (Pingali 2012). Economically, the high cost of synthetic fertilizers places a heavy burden on farmers, especially smallholders in developing countries. Fertilizers can constitute up to 30% of total production costs for small‐scale farmers, often forcing them into debt cycles due to the need for loans to purchase these inputs (Biru, Zeller, and Loos 2020; Bonilla Cedrez et al. 2020; Dhillon and Moncur 2023). This financial strain undermines the economic stability and sustainability of smallholder farming systems.

The environmental impact of synthetic fertilizer use is equally concerning. Long‐term application has led to soil degradation, decreasing soil organic matter and fertility, which in turn reduces crop yields and increases farmers' reliance on chemical inputs (Mueller et al. 2012; Zhang et al. 2015). Studies indicate that the continuous use of these fertilizers disrupts soil microbial communities essential for maintaining soil health (Lori et al. 2017). This disruption negatively impacts nutrient cycling and soil structure, further exacerbating soil degradation (Galloway et al. 2003).

Moreover, fertilizer runoff is a significant contributor to water pollution. Excess nutrients, particularly nitrogen and phosphorus, from agricultural fields are washed into water bodies, causing eutrophication and harmful algal blooms (Carpenter, Bennett, and Peterson 2006). These blooms deplete oxygen in the water, creating hypoxic zones or “dead zones” that severely impact aquatic life and biodiversity (Diaz and Rosenberg 2008). Agricultural runoff is a major global source of water pollution, affecting both freshwater and marine ecosystems (Galloway et al. 2003).

Synthetic fertilizers also significantly contribute to greenhouse gas emissions. The production and application of nitrogen fertilizers result in the release of nitrous oxide (N_2_O), a potent greenhouse gas with a global warming potential much higher than that of CO_2_ (Shukla et al. 2019). Agricultural N_2_O emissions have increased by over 30% since the 1980s, driven largely by the increased use of synthetic fertilizers (Davidson 2009). This rise in emissions exacerbates climate change, contributing to global temperature increases and associated climatic disruptions.

In response to these challenges, sustainable alternatives to synthetic fertilizers are being explored. Organic farming practices, which use compost, manure, and other organic inputs, have been shown to improve soil health and reduce environmental pollution (Reganold and Wachter 2016). Organic farming can enhance soil organic carbon levels, improve water retention, and support biodiversity, making it a viable alternative for sustainable agriculture (Seufert, Ramankutty, and Foley 2019). Precision agriculture technologies, which optimize fertilizer application based on soil and crop needs, offer another promising solution. These technologies can significantly reduce fertilizer use, minimizing environmental impacts while maintaining crop yields (Gebbers and Adamchuk 2010).

Biofertilizers, which employ microbial inoculants to enhance nutrient availability to plants, represent another sustainable alternative. Biofertilizers can improve nutrient uptake, increase crop yields, and enhance soil health without the adverse environmental effects associated with synthetic fertilizers (Vessey 2003). The prompt and appropriate supplementation of essential nutrients is a critical factor influencing plant height. Biofertilizers can significantly boost plant height by ensuring that plants receive sufficient macro and micronutrients from the soil (Yilmaz and Karik 2022; Yilmaz 2022). However, the adoption of these sustainable practices and technologies faces several barriers, including higher initial costs, lack of access to knowledge and resources, and the need for supportive policy frameworks (Pretty, Williams, and Toulmin 2012).

While synthetic fertilizers have significantly boosted agricultural productivity, their economic and environmental costs necessitate a shift towards more sustainable practices. Promoting organic farming, precision agriculture, and biofertilizers can mitigate the adverse effects of synthetic fertilizers, enhance soil health, and support long‐term agricultural sustainability (Pingali 2012). Therefore, integrating these sustainable practices into mainstream agriculture is imperative for achieving a balanced approach that maintains productivity while preserving environmental integrity. Future research and policy efforts should focus on overcoming the barriers to adoption, such as higher initial costs and lack of access to knowledge and resources, to ensure a widespread and effective transition towards sustainable agricultural systems.

## 7 Long‐Term Impacts on Food Security and Health

According to data from the Global Nutrition Report (2021), malnutrition remains a critical challenge in regions such as South Asia and Sub‐Saharan Africa. Despite the increase in food production, stunting rates among children under five remain high, with 31.7% in South Asia and 33.1% in Sub‐Saharan Africa (Development Initiatives 2021). This indicates that although food availability has improved, the nutritional quality of diets has not kept pace, leading to continued health challenges.

Micronutrient deficiencies, such as iron, vitamin A, and zinc, are widespread in regions impacted by the Green Revolution. For instance, the consumption of polished rice, which is low in these essential nutrients, has been linked to high rates of anemia and stunting among children (Muthayya et al. 2013). These deficiencies contribute to child morbidity and mortality, while also negatively affecting cognitive development, which can hinder educational outcomes and future earning potential (Dewey and Begum 2011; Black et al. 2013). Efforts to mitigate these deficiencies include biofortification programs that enrich staple crops with essential nutrients. For example, biofortified rice and maize varieties that are rich in iron and zinc have demonstrated promise in improving nutrient intake and addressing these deficiencies (Bouis and Saltzman 2017; Saltzman et al. 2013).

In addition to agricultural interventions, educational programs are essential in promoting dietary diversity and nutrition awareness. Public health campaigns aimed at educating communities on the importance of balanced diets can shift consumption patterns away from staple crop reliance (Herforth and Ahmed 2015). Schools and community programs also play a pivotal role in fostering long‐term dietary changes, ensuring that both children and their families understand the value of consuming a variety of nutrient‐rich foods (Food and Agriculture Organization of the United Nations 2021; Dudley, Cotton, and Peralta 2015).

Sustainable agricultural practices such as crop rotation, intercropping, and agroforestry are vital for improving soil health and food supply diversity. These practices not only enhance productivity but also support the creation of more nutritious diets, thereby addressing both food security and long‐term health outcomes (Kremen and Miles 2012). Economic stability and market access are also critical factors in ensuring that increased agricultural productivity translates into improved food security. Recent economic shocks, such as the global financial crisis and the COVID‐19 pandemic, have severely impacted vulnerable populations, making it essential to implement policies that support economic resilience and food system stability (Headey and Ruel 2022).

## 8 Case Studies and Real‐World Examples

The global nature of hidden hunger and its multifaceted impacts are well‐illustrated through diverse geographical case studies. Each region faces unique challenges and has developed specific strategies to combat micronutrient deficiencies and improve food security. These examples provide a balanced view of both successes and ongoing challenges.

In India, the Green Revolution significantly increased the production of staple crops, but it also led to reduced dietary diversity and persistent micronutrient deficiencies. Despite higher yields, many Indian households continue to suffer from iron and vitamin A deficiencies due to overreliance on rice and wheat (Narayanan and Gerber 2017). To address this, India has implemented large‐scale biofortification programs. For instance, the introduction of iron‐rich pearl millet has shown promising results in improving iron status among rural populations (Bouis and Saltzman 2017).

Biofortification efforts in South Africa have introduced fortified maize and orange‐fleshed sweet potatoes (OFSP), significantly reducing anemia and vitamin A deficiencies among children (Siwela et al. 2020; Kiran et al. 2022). Similarly, in Zambia, the introduction of OFSP, rich in beta‐carotene, has improved vitamin A intake among children, reducing deficiency‐related health issues (Lividini and Fiedler 2015).

In Guatemala, a multisectoral approach combining agricultural interventions with nutrition education and health services has reduced stunting in targeted communities (Tschida et al. 2021; Isaac et al. 2023). Additionally, maternal education programs have significantly improved child nutrition. Similarly, in Bolivia, the introduction of iron‐ and zinc‐biofortified potatoes has improved the nutritional status of rural populations in the Andean highlands, where iron and zinc deficiencies are prevalent (International Potato Center 2022). Latin America presents further examples of how biofortification and multisectoral approaches are used to combat hidden hunger.

In Southeast Asia, the Philippines has tackled hidden hunger through its School‐Based Feeding Program, providing fortified meals that have improved the nutritional status and academic performance of schoolchildren (Agujar, Villanueva, and Santos 2020). In Latin America, Brazil's Zero Hunger Program (Fome Zero) offers another successful approach. The program has integrated social protection policies with agricultural support and nutrition education to significantly reduce hunger and malnutrition (Bither‐Terry 2016). Additionally, in 2021, the World Food Programme piloted a similar school feeding program in Maguindanao (Philippines) using iron‐fortified rice, significantly improving iron intake and reducing anemia rates among schoolchildren (World Food Programme 2021).

In contrast, some regions continue to struggle with the dual burden of malnutrition and obesity. Mexico, for example, faces high rates of both undernutrition and overweight, particularly among children. The complex interplay between socioeconomic factors and dietary patterns has led to this dual burden (Rivera et al. 2014). Mexico's response includes implementing sugar‐sweetened beverage taxes and front‐of‐package labeling to promote healthier dietary choices. Although these measures have shown some success in reducing sugary drink consumption, ongoing efforts are needed to address broader dietary issues and improve overall nutrition.

Ethiopia's Productive Safety Net Program has improved food security and dietary diversity through food and cash transfers, coupled with agricultural support and nutrition education (Araya 2020). This program illustrates the importance of combining short‐term relief with long‐term development strategies to combat hidden hunger. The program's integration of agricultural support, such as providing seeds and tools, with nutrition education has been key to its success.

These case studies highlight the global nature of hidden hunger, and the diverse strategies employed to address it. From biofortification in India and Zambia to multisectoral approaches in Guatemala and Brazil, innovative solutions are being implemented worldwide. However, persistent challenges remain, particularly in regions facing the dual burden of malnutrition and obesity. Comprehensive, context‐specific strategies that integrate agricultural, nutritional, and educational interventions are essential for addressing hidden hunger and improving global food security and health outcomes (Fanzo et al. 2018).

## 9 Towards a Sustainable Agricultural Future

The pursuit of sustainable agriculture has gained momentum in recent years as the global community recognizes the need to balance food production with environmental stewardship and human health (Tilman et al. 2017). Cutting‐edge research in this field is increasingly interdisciplinary, integrating agronomy, nutrition, and environmental science to develop holistic solutions. Sustainable agricultural practices are essential for meeting the food needs of a growing population while mitigating environmental impacts (Shafi et al. 2019).

Recent developments in sustainable agriculture have focused on improving crop yields through eco‐friendly methods. One promising area is the use of precision agriculture technologies, which employ data analytics, sensors, and satellite imagery to optimize farming practices. Precision agriculture can significantly reduce the use of water, fertilizers, and pesticides, thereby decreasing the environmental footprint of farming operations (Shafi et al. 2019). These technologies enable farmers to apply inputs more efficiently, improving crop productivity and resource use efficiency.

Interdisciplinary approaches are crucial in advancing sustainable agriculture. Integrating agronomy with nutrition science has led to the development of biofortified crops that address both food security and micronutrient deficiencies. Biofortified varieties of rice, wheat, and maize have been enriched with essential vitamins and minerals such as vitamin A, iron, and zinc. These biofortified crops can improve nutritional outcomes without compromising yield (Bouis and Saltzman 2017). This approach not only enhances dietary quality but also supports the health of vulnerable populations.

Environmental science plays a critical role in shaping sustainable agricultural practices. Research on soil health and conservation agriculture has shown that techniques such as no‐till farming, cover cropping, and crop rotation can improve soil fertility and reduce. Maintaining healthy soils is fundamental to sustainable agriculture, as it enhances carbon sequestration, water retention, and biodiversity. These practices contribute to long‐term agricultural productivity and resilience against climate change (Finney, White, and Kaye 2016; Büchi et al. 2017; Greene, Broyles, and Pearson 2024).

Policymakers have a pivotal role in promoting sustainable agriculture. Implementing policies that incentivize environmentally friendly practices and support smallholder farmers is essential. Governments should provide subsidies for sustainable farming inputs, such as organic fertilizers and biofertilizers, and invest in agricultural research and extension services (Pretty, Williams, and Toulmin 2012). Additionally, policies that promote market access for sustainably produced goods can enhance the economic viability of these practices for farmers.

Educational and extension programs are vital for disseminating knowledge about sustainable agricultural practices. Training farmers in the use of precision agriculture technologies and sustainable farming techniques can enhance adoption rates. Integrating nutrition education into agricultural extension services can also improve dietary outcomes (Herforth and Ahmed 2015). These programs should be tailored to local contexts and involve community participation to ensure relevance and effectiveness.

Collaboration between researchers, practitioners, and policymakers is necessary to advance sustainable agriculture. Interdisciplinary research initiatives that bring together experts from agronomy, nutrition, and environmental science can generate innovative solutions. The CGIAR Research Program on Climate Change, Agriculture, and Food Security focuses on developing climate‐smart agricultural practices that enhance resilience and productivity (Vermeulen, Campbell, and Ingram 2012). Such collaborations can help bridge the gap between scientific research and practical implementation.

Sustainable agricultural practices must also address social equity and inclusivity. Ensuring that smallholder farmers, women, and marginalized groups have access to resources and knowledge is crucial for achieving food security and sustainability (Fanzo et al. 2018). Inclusive policies that support diverse farming systems and recognize the contributions of small‐scale farmers to food production are essential. Empowering these groups through targeted interventions can enhance the overall impact of sustainable agriculture initiatives.

**FIGURE 5:**
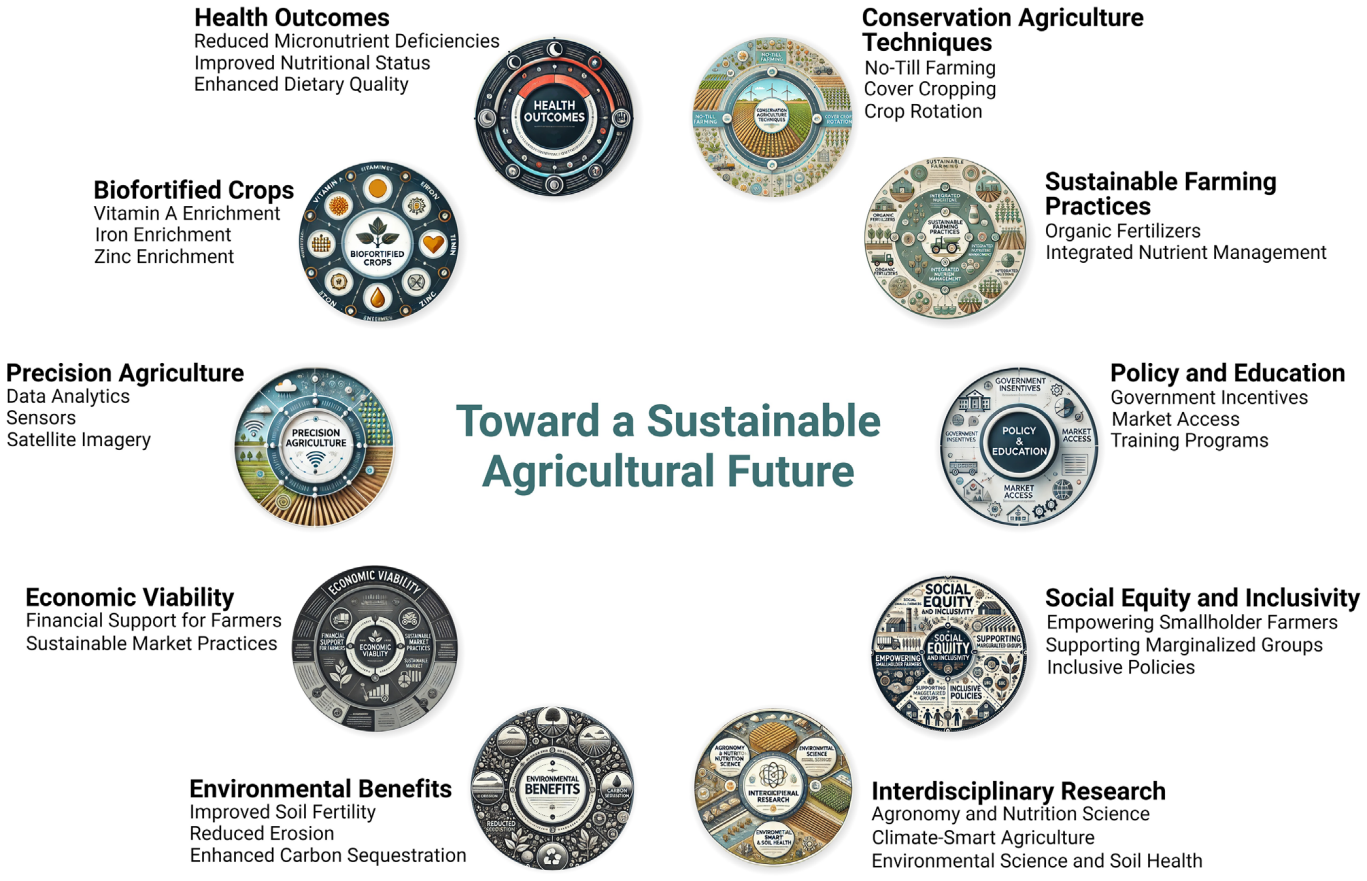
Key components and supporting elements of sustainable agriculture.

Advancing sustainable agriculture requires a multifaceted approach that integrates cutting‐edge research, interdisciplinary collaboration, and supportive policies. Precision agriculture, biofortification, and conservation agriculture are promising areas that can enhance food security while mitigating environmental impacts. Policymakers, researchers, and practitioners must work together to create an enabling environment for sustainable agricultural practices. By adopting these strategies, we can move towards a future where agriculture is productive, resilient, and environmentally sustainable (Tilman et al. 2017). Figure 5 highlights the core elements and strategies for sustainable agriculture, including biofortification, precision agriculture, and conservation practices. This visual supports the discussion on the multifaceted approach required to ensure agricultural sustainability while addressing economic, environmental, and social challenges.

## 10 Conclusion

The comprehensive review of the Green Revolution and its far‐reaching impacts highlights a dual narrative of success and challenges. While the Green Revolution significantly increased global food production, contributing to substantial reductions in hunger, it also led to unintended consequences that continue to affect food security and public health. The shift towards HYVs of staple crops has resulted in decreased nutritional quality, dietary monocultures, and environmental degradation.

The persistence of hidden hunger, characterized by micronutrient deficiencies despite adequate caloric intake, underscores the limitations of focusing solely on crop yields. Nutritional deficiencies remain prevalent, particularly in regions that heavily rely on staple crops like rice and wheat. Addressing these deficiencies requires a multifaceted approach that integrates agronomic innovations, nutritional interventions, and environmental sustainability.

Improved agricultural practices are crucial for enhancing both the quantity and quality of food production. Precision agriculture technologies, biofortified crops, and conservation agriculture offer promising solutions to increase yields while maintaining or enhancing nutrient content. These practices can reduce the environmental footprint of farming, improve soil health, and support long‐term agricultural sustainability.

Dietary diversity is essential for overcoming hidden hunger and improving overall health outcomes. Furthermore, it is essential to acknowledge the bidirectional relationship between consumer preferences and agricultural practices. Consumer demand plays a critical role in shaping the types of crops cultivated, as market forces influence the prioritization of certain staple crops over others. This dynamic often leads to the widespread adoption of high‐yield, low‐nutrient varieties, which, while contributing to food security, can exacerbate issues like hidden hunger. In turn, agricultural practices and available food options can shape consumer habits and dietary patterns, creating a feedback loop that either reinforces or mitigates micronutrient deficiencies. Future efforts to combat hidden hunger must consider this complex interaction, promoting both agricultural diversity and consumer awareness of nutrient‐rich foods. Promoting the cultivation and consumption of a wide variety of nutrient‐dense foods can help address micronutrient deficiencies and reduce the incidence of diet‐related NCDs. Educational initiatives and public health campaigns are vital for raising awareness about the importance of a balanced diet and fostering healthier eating habits.

Policy initiatives play a pivotal role in creating an enabling environment for sustainable agriculture and improved nutrition. Governments and international organizations must implement policies that support sustainable farming practices, provide incentives for the adoption of biofortified crops, and ensure access to diverse and nutritious foods. Integrating nutrition education into agricultural extension services and community programs can enhance the impact of these policies.

The case studies from various regions illustrate both the successes and challenges in addressing hidden hunger and promoting sustainable agriculture. These examples demonstrate the effectiveness of integrated approaches that combine agricultural, nutritional, and educational interventions. However, they also highlight the need for continued efforts and innovation to overcome persistent challenges and adapt to changing environmental and socio‐economic conditions.

Addressing the complex issues of hidden hunger and food security requires a holistic approach encompassing improved agricultural practices, dietary diversity, and supportive policies. By integrating these strategies, we can build resilient and sustainable food systems that not only increase food production but also enhance nutritional outcomes and environmental health. The journey towards a sustainable agricultural future is ongoing, and it necessitates the collaboration of researchers, policymakers, practitioners, and communities to ensure that the benefits of the Green Revolution are realized in a balanced and equitable manner.

## Author Contributions

**Hilal Yilmaz:** conceptualization (lead), investigation (lead). **Abdurrahim Yilmaz:** visualization (lead), writing – original draft (lead), writing – review and editing (lead).

## Conflicts of Interest

The authors declare no conflicts of interest.

## Data Availability

The data generated during this study are available from the corresponding author upon reasonable request.
